# A computational study of gene expression patterns in head and neck squamous cell carcinoma using TCGA data

**DOI:** 10.1080/20565623.2024.2380590

**Published:** 2024-08-14

**Authors:** Saqib Rauf, Sami Ullah, Muhammad Adil Abid, Asad Ullah, Gullzar Khan, Ainee Urooj Khan, Gulzar Ahmad, Muhammad Ijaz, Sidra Ahmad, Sulaiman Faisal

**Affiliations:** 1 Institute of Integrative Biosciences, CECOS University Peshawar, 25000, Pakistan; 2 Institute of Biotechnology & Genetic Engineering, University of Agriculture Peshawar, 25000, Pakistan; 3 Centre for Omics Sciences, Islamia College Peshawar, 25000, Pakistan

**Keywords:** Big Data, DEG, Gene Ontology, HNSCC, *KRT13*, TCGA

## Abstract

**Aim:** Head and Neck squamous cell carcinoma (HNSCC) is the second most prevalent cancer in Pakistan. **Methods:** Gene expression data from TCGA and GETx for normal genes to analyze Differentially Expressed Genes (DEGs). Data was further investigated using the Enrichr tool to perform Gene Ontology (GO). **Results:** Our analysis identified most significantly differentially expressed genes and explored their established cellular functions as well as their potential involvement in tumor development. We found that the highly expressed Keratin family and *S100A9* genes. The under-expressed genes *KRT4* and *KRT13* provide instructions for the production of keratin proteins. **Conclusion:** Our study suggests that factors such as poor oral hygiene and smokeless tobacco can result in oral stress and cellular damage and cause cancer.

## Plain Language Summary

The Cancer Genome Atlas (TCGA) holds vast cancer data processed with powerful computers and cloud tech. This sparks new bioinformatics for better cancer diagnosis, treatment, and prevention. In Southeast Asia, Head and Neck Squamous Cell Carcinoma (HNSCC) is prevalent. We used TCGA and GETx data to study gene expression. High-expression Keratin and *S100A9* genes fight cellular damage under stress, while under-expressed *KRT4 and KRT13* genes shape cell structure. Poor oral care and smokeless tobacco could induce cell damage, sparking cancer mutations. Unveiling HNSCC mechanisms may guide targeted treatments and preventive strategies.

## Abstract (plain language summary)

The Cancer Genome Atlas (TCGA) stores, shares, and analyzes extensive cancer data using high-performance computers and clouds.

New bioinformatics tools facilitating analysis of large data sets to enhance cancer diagnosis, treatment, and prevention.

Head and neck squamous cell carcinoma (HNSCC) accounts for 8–10% of Southeast Asia's cancer cases and is Pakistan's second most prevalent cancer.

Gene expression data from TCGA (tumor genes) and GETx (normal genes) were used to study Differential Expressed Genes (DEGs).

Enrichr tool and Gene Ontology (GO) enrichment analysis investigated candidate DEGs.

Highly expressed Keratin family and *S100A9* genes combat cellular damage, upregulated during stress.

Under-expressed *KRT4 and KRT13* genes instruct keratin protein production for epithelial cell structure.

Poor oral hygiene and smokeless tobacco could lead to oral stress, cell damage, mutations and oncogenesis.

Understanding HNSCC mechanisms aids targeted therapies and prevention strategies.

## Graphical Abstract

**Figure:**
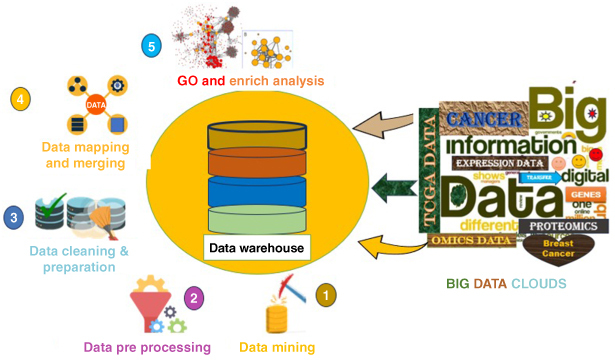

Bioinformatics is an integrative field which is the interaction between computer science and biology. With the advancement in the technologies, genomes of many species have been completely sequenced and stored [1,2]. In the era of big data transformation of complex data into valuable knowledge becomes more and more important and Bioinformatics and machine learning methodologies play a significant part in extracting knowledge out of this complex data [3]. There are various databases including DNA, RNA, Protein, expression and disease databases and they all are publicly accessible online [4,5]. Cancer is considered a usual cause of patient death in the clinic because of poor diagnoses and a general lack of therapies with good outcomes. Computational tools can be applied to improve diagnosis and therapies of the tumor and we can identify and validate biomarkers in early stages of cancer [6–8]. With the aid of new technologies and methods we can study and predict the efficiency and effectiveness of existing drugs, ushering in a the era of Precision Medicine [9,10].

Squamous cells can be found on the outer layer of the skin in the mucous membrane, these are the moist tissues which lines airways and intestines. Cancer in mouth, nose and throat and other regions of the head falls under the Head and Neck Squamous Cell Carcinoma or HNSCC [11], it can spread into lymph nodes or lungs and it is the considered as the sixth most common cancer worldwide [12,13]. In the last few years in Pakistan, HNSCC has ranked as the second most prevalent cancer because of its rising incidence. Smokeless tobacco, such as moist snuff (naswar) and betel leaf (pan), Areca nut (gutka) and poor oral hygiene are major risk factors which can cause chronic inflammation such as periodontitis and may contribute to oncogenesis [14–17].

Differentially expressed genes (DEGs) analyses means finding out quantitative changes in expression levels between experimental groups after taking out the normalized read count data and performing statistical analysis. Rapaport *et al.* mentioned three steps in their study for differential gene expression analysis using RNA-Seq data: normalization of counts, parameter estimation of the statistical model and tests for differential expression [18–20]. Normalization is basically needed to check the difference in sequencing depths so that this data can be directly compared with different samples. Normalization is also applied to correct other effects such as variations in GC content and transcript length [18]. TCGA data are normalized by Reads per Kilo base per Million mapped reads (RPKM) approach. Normalization is dividing each read count by total read count in its sample. There are a few reasons why normalization is important, the first reason is that it has been found that it has a great impact on the results of the analysis [21,22]. The second understanding is that, for example a few genes are highly expressed in one condition so because of this it will have a greater share of the total molecules and a smaller fraction of reads will be left for the remaining genes. This will cause false results of differential expression for the non-differential expression genes [23–26].

Genotype-Tissue Expression (GETx) database stores and curates data of healthy individuals to examine the relationship between genetic variants and gene expression. All datasets are available on the GETx data portal and it is freely available for public. Statistical data on the GETx data portal is approximately 11688 RNA-Seq samples across nearly 54 tissue sites from about 1000 individuals [27,28]. Normal gene expression data used in this work are from GETx and normalized in a Transcripts Per Million (TPM). Gene Ontology (GO) project was initiated in 1998 with only three model organism databases. It has three major goals, and the most important one was to develop a controlled, structured vocabularies known as ontologies, second was to apply these ontology terms in the annotation of sequences, genes or gene products in biological databases and the final one was to provide this data and software tools developed for GO data to the public [29,30]. We used Enrichr tool of the Ma'ayan Laboratory for GO enrichment analysis in which our set of genes was assigned with ontologies from the GO database.

## Materials & methods

### Data collection

The gene expression data for Head and Neck Squamous Cell Carcinoma (HNSCC) was obtained from The Cancer Genome Atlas (TCGA) database. TCGA is a publicly available database that contains multi-omics data for various types of cancer, including HNSCC. The HNSCC dataset included RNA-sequencing data from 528 tumor samples and 43 matched normal tissue samples. Total 80 tumor and normal samples were used to identify differentially expressed genes.

### Data preprocessing

Transcriptome profiling data of HNSCC was downloaded from TCGA data portal dated July 2022. This data was stored in TXT file and was normalized in FPKM. Data was then organized and cleaned in Excel 2016. The data in excel file from TCGA contained Ensembl ID's and expression values. For mapping, a python program was developed and mapping table was downloaded from Ensembl Database. Normal gene expression data in TPM of Esophagus – Mucosa data was downloaded from GDC data portal dated July 2022. This data was stored in a GCT zip file and was then organized in an Excel file. Using python program the expression values of tumor data and normal data was mapped according to Ensembl ID's.

### Data cleaning & preparation

The data was filtered to remove genes with low expression levels and samples with poor sequencing quality. Normalization was performed using the FPKM method in the python program to remove technical variations [31]. Excel 2013 was used to clean and prepare this complex data from TCGA and GETx the total 500 DEG samples were downloaded for python program and to find out the differences, standard deviation and fold changes between the data.

### Data mapping & merging

**Figure 1.**
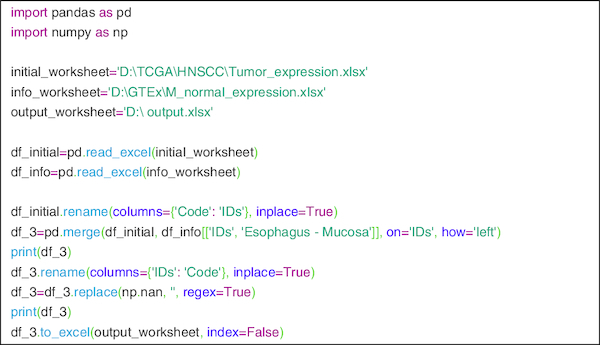
It show the code of python through which the data are processed.

To develop a program for mapping Ensemble ID's with gene ID's and to compare tumor data with normal expression data Python latest version 3.7.4 was used and for Integrated Development Environment (IDE) ‘PyCharm community edition 2019.1.3’ of ‘JetBrains’ was used. Merge function of ‘Pandas package version 0.24.2’ was used for comparing tumor data with normal data and for array processing ‘numpy package version 1.16.4’ was used. The code segment is also shown in Figure 1. The code is only used to compare tumor expression and normal expression data from two different excel files according to the ID's.

### Conversion of FPKM into TPM

For conversion of FPKM into TPM the following equation was used [31]:

$${\text{TPM}}_{i}=\left(\frac{{\text{FPKM}}_{i}}{\sum_{j}{\text{FPKM}}_{j}}\right)\times 10^{6}$$

Using this equation, FPKM tumor data was converted into TPM so it can easily be compared with normal data.

### GO enrichment analysis

For enrichment analysis Enrichr tool of Ma'ayan Laboratory [29,32] was used to find out the molecular function, biological function and cellular components of the resultant genes. Enrichr tool takes a set of genes and provide you with ontologies, pathways, transcriptions and cell type biological processes, molecular functions, cellular components, and signaling pathways associated with the DEGs [33]. The adj P-value threshold for selecting the significant go or pathway is 0.05.

### Network analysis

The protein-protein interaction (PPI) network for the DEGs was constructed using the Search Tool for the Retrieval of Interacting Genes/Proteins (STRING) database. The threshold value which is used for string is 0.90. The network was visualized using Cytoscape software. The hub genes in the network were identified based on their degree of connectivity [34].

### Statistical analysis

Statistical analyses in this study were conducted using SPSS software. To compare the two groups, a two-tailed *t*-test was employed. Statistical significance was set at p < 0.05.

## Results

### Overexpressed & under expressed genes

**Figure 2.**
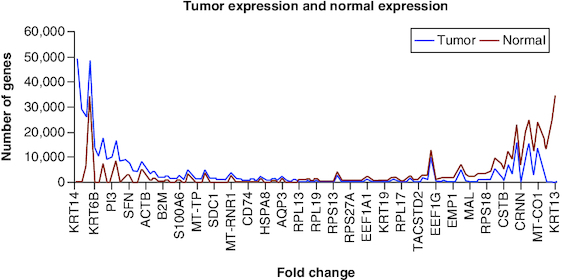
Graphical comparison of both expression data's, on the left side tumor data is highly expressed, whereas on the right side of the graph normal data is highly expressed and in the middle the expression is similar.

**Figure 3.**
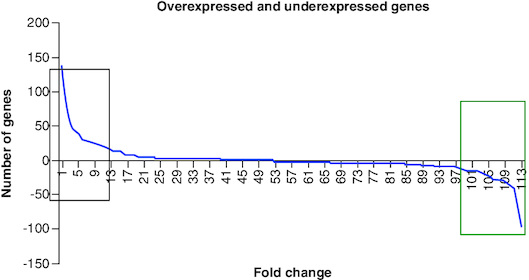
The graphical representation of the genes shows overexpressed genes in the green box on the right side, with a 5–140-fold change. The left box contains under expressed genes, while the middle box includes genes with similar fold changes.

From our finds we have found 52 over expressed genes and 61 under expressed genes which are shown in Tables 1 & 2 and in Figure 2 below. From DEG we have got 2–140-fold change difference in over expression genes and (-2) – (-140) fold change difference in under expressed genes which are shown in Figure 3. The resultant genes were then divided into three groups according to their fold changes. In Group I, we have selected those genes that have (2–20), Group II (20–40) and in Group III (40–140) fold change.

**Table 1.** This table shows a detailed list of overexpressed genes and under expressed genes.

Fold change	Number of genes	Overexpressed genes
Group I (40–140)	4	*KRT14*, *KRT16*, *KRT6A*, *S100A9*
Group II (20–40)	7	*KRT6B*, *S100A7*, *KRT5*, *KRT17*, *PI3*, *MT-RNR2*, *KRT6C*
Group III (2–20)	41	*SPRR1B*, *SFN*, *FABP4*, *KRT1*, *SPRR1A*, *ACTB*, *GJB2*, *TMSB10*, *SLC25A5*, *B2M*, *JUP*, *CALML5*, *PKP1*, *S100A6*, *FSCN1*, *MT-ND5*, *GAPDH*, *MT-TP*, *IFI6*, *MT-ND6*, *DSP*, *SDC1*, *IGFL1*, *KRTDAP*, *MMP1*, *MT-RNR1*, *HLA-E*, *IGHA1*, *MSN*, *CD74*, *HLA-C*, *MAGEA4*, *PERP*, *HSPA8*, *HLA-DRA*, *HLA-B*, *LGALS3BP*, *AQP3*, *BCAP31*, *IGKC*, *PFN1*

**Table 2.** This table shows a detailed list of under expressed genes.

Fold changes	Number of Genes	Under expressed genes
Group I (-40) – (-140)	2	*KRT4*, *KRT13*
Group II (-20) – (-40)	8	*MT-CO3*, *CRNN*, *MT-ND4*, *MT-CO2*, *MT-ATP8*, *MT-CO1*, *MT-ATP6*, *SPRR3*
Group III (-2) – (-20)	51	*RPL13*, *FTH1*, *RPS25*, *PPL*, *RPL19*, *KRT15*, *RPS7*, *RPSA*, *RPS1*, *HSPB1*, *RPL9*, *RPL14*, *RPS27A*, *SLURP1*, *ECM1*, *CRCT1*, *EEF1A1*, *RPL5*, *MUC21*, *CRABP2*, *KRT19*, *SPINK5*, *RPLP0*, *RPL3*, *RPL17*, *RPS16*, *CNFN*, *CD24P4*, *TACSTD2*, *RPL13A*, *RPS12*, *S100A8*, *EEF1G*, *IL1RN*, *TPT1*, *LY6D*, *EMP1*, *RPLP1*, *MT-ND3*, *ANXA1*, *MAL*, *TGM3*, *S100A14*, *CSTA*, *RPS18*, *RHCG*, *MT-ND1*, *MT-ND4L*, *CSTB*, *MT-CYB*, *MT-ND2s*

### Group I overexpressed genes

**Figure 4.**
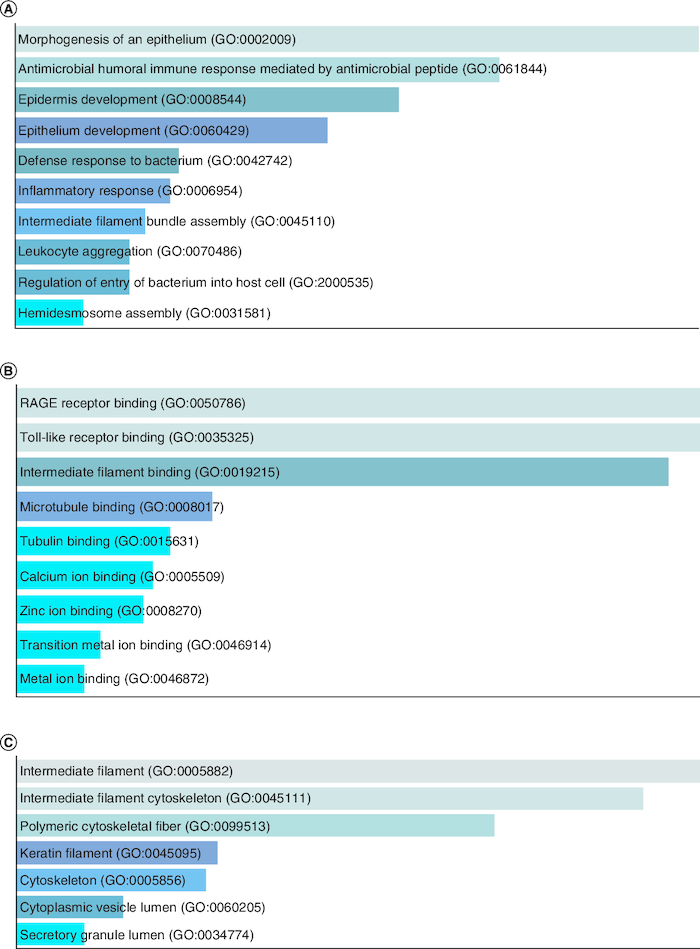
GO enrichment analysis of Group I overexpressed genes in tumor data. **(A)** GO biological process, **(B)** GO molecular function and **(C)** GO cellular component.

Group I comprises of 4 overexpressed genes in tumor cells, including members of the keratin gene family, which are intermediate filament-forming proteins that provide mechanical and non-mechanical stress protection in epithelial cells. Keratins have been reported as highly expressed in HNSCC and are subject to post-transcriptional modifications, with phosphorylation serving as a major regulator [35]. Keratins have potential applications in cancer diagnosis and as prognostic indicators in various epithelial malignancies [36]. The S100A9 gene in Group I is a calcium-binding protein that is normally expressed in myeloid cells but is upregulated in inflammation and cancer. Due to its multiple ligands and post-translational modifications, it plays a role in the initial growth of cancer and is involved in inflammatory events [21]. Figure 4A GO biological process, Figure 4B GO molecular function and Figure 4C GO cellular component.

### Group II overexpressed genes

**Figure 5.**
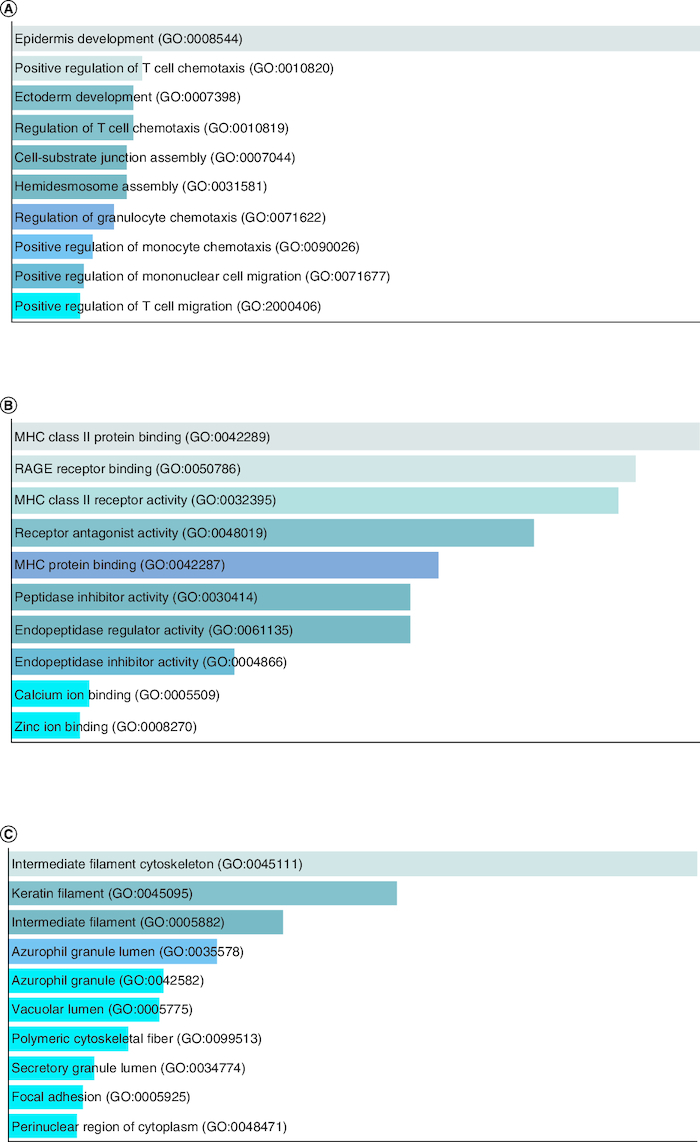
GO enrichment analysis of Group II overexpressed genes in tumor data. **(A)** GO biological process, **(B)** GO molecular function and **(C)** GO cellular component.

The fold change of Group II overexpressed genes ranges from 20–40, comprising 7 genes, some of which belong to the keratin family, as explained in the overexpressed genes of Group I. Other genes in this group include S100A7, P13 and MT-RNR2. S100A7 plays a role in the initial stages of cervical tumor progression [37]. The function of P13 gene depends on its location, producing reactive oxygen species in mitochondria to control cell turnover, while reducing the expression of both HTLV-1 and Tax-controlled cellular genes in the nuclear region [37]. In cancerous cells, mitochondrial genes MT-RNR2 and MT-CYB are significantly mutated, as studies have shown the need for more energy for reproduction [38]. Figure 5A GO biological process, Figure 5B GO molecular function and Figure 5C GO cellular component.

### Group III overexpressed genes

**Figure 6.**
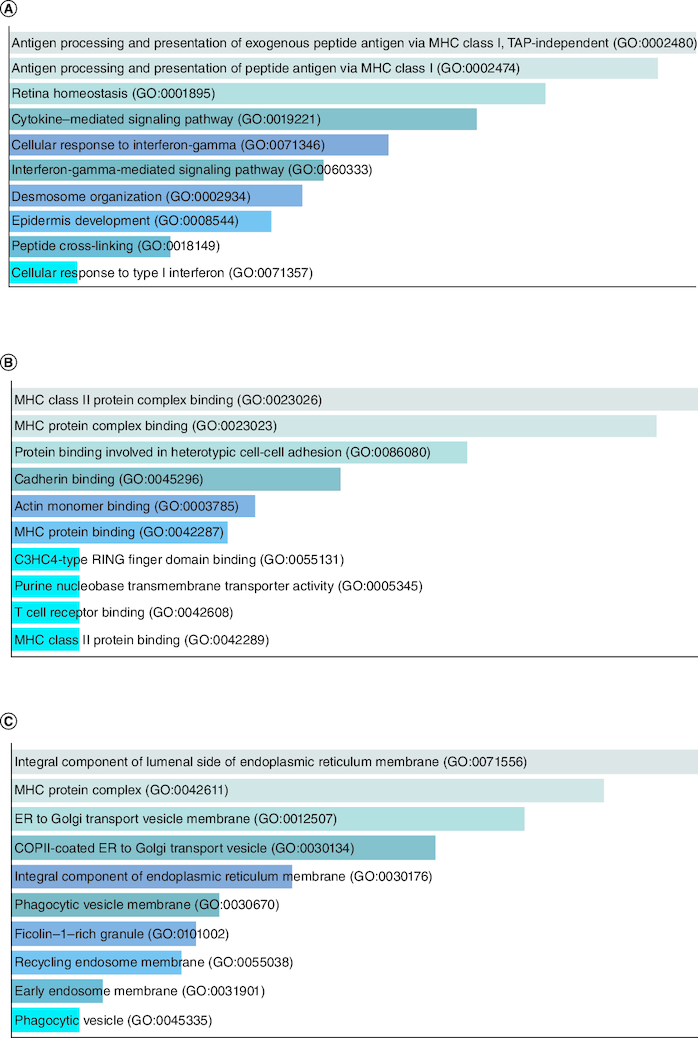
GO enrichment analysis of Group III overexpressed genes in tumor data. **(A)** GO biological process, **(B)** GO molecular function and **(C)** GO cellular component.

Fold change of Group III overexpressed genes is 2–20 and there are 41 genes in this group. This group includes genes from keratin family, mitochondrial genes. Some of these genes are biomarkers for squamous metaplasia [39], SFN genes help in cell death in cancer cells via apoptosis [40]. Figure 6A GO biological process, Figure 6B GO molecular function and Figure 6C GO cellular component.

### Group I under expressed genes

**Figure 7.**
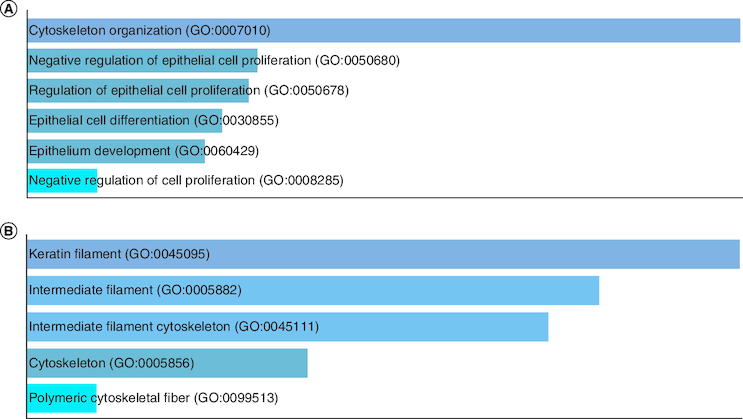
GO enrichment analysis of Group I under expressed genes in tumor data. **(A)** GO biological process and **(B)** GO cellular component.

The group I contain only two genes *KRT4 and KRT13* which are under expressed in tumor data and over expressed in normal data. Deregulation of *KRT4 and KRT13* genes is associated with impaired epithelial differentiation and organization during tumor progression, and they are normally expressed in the oral cavity [41]. Figure 7A GO biological process, Figure 7B GO cellular component.

### Group II under expressed genes

There are eight genes in Group II under expressed genes *MT-CO3, MT-CO2, MT-CO1, CRNN, MT-ND4, MT-ATP8, MT-ATP6 and SPRR3. MT-CO3, MT-CO2, MT-CO1* genes make complex IV, which is a third and final enzyme of electron transport chain. A study has shown that by down regulation of miR-5787 this will inhibit translation of *MT-CO3* genes which will enhance glycolysis and in result more lactate will produce and cisplatin will loss efficacy in a low-pH environment and will develop cisplatin resistance [42]. Cisplatin is a drug used in the treatment of cancer. These genes are generally under expressed in strongly mtDNA- and mtRNA-depleted cancer types [43]. CRNN (Cornulin) gene has a strong tumor suppressive ability in which its arrest cell cycle at G1/S checkpoint in Esophagus squamous cell carcinoma (ESCC) but it has been studied that it is down regulated in tumor tissues without specific pathogenic mutation, but it is appeared that it interact with tobacco smoking that contribute to the risk of developing HSCC [44].

**Figure 8.**
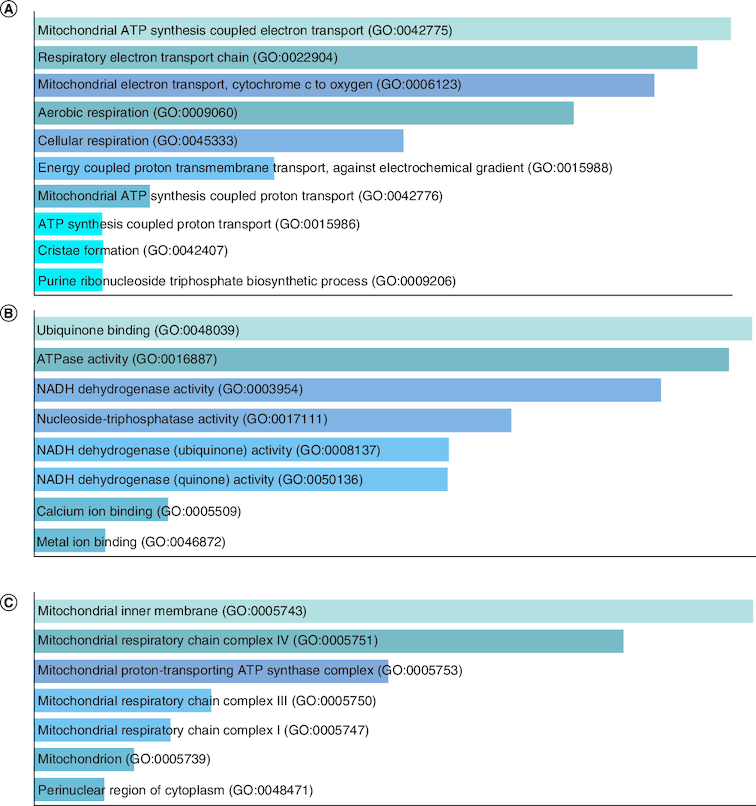
GO enrichment analysis of Group II under expressed genes in tumor data. **(A)** GO biological process, **(B)** GO molecular function and **(C)** GO cellular component.

Figure 8A GO biological process, Figure 8B GO molecular function and Figure 8C GO cellular component.

### Group III under expressed genes

**Figure 9.**
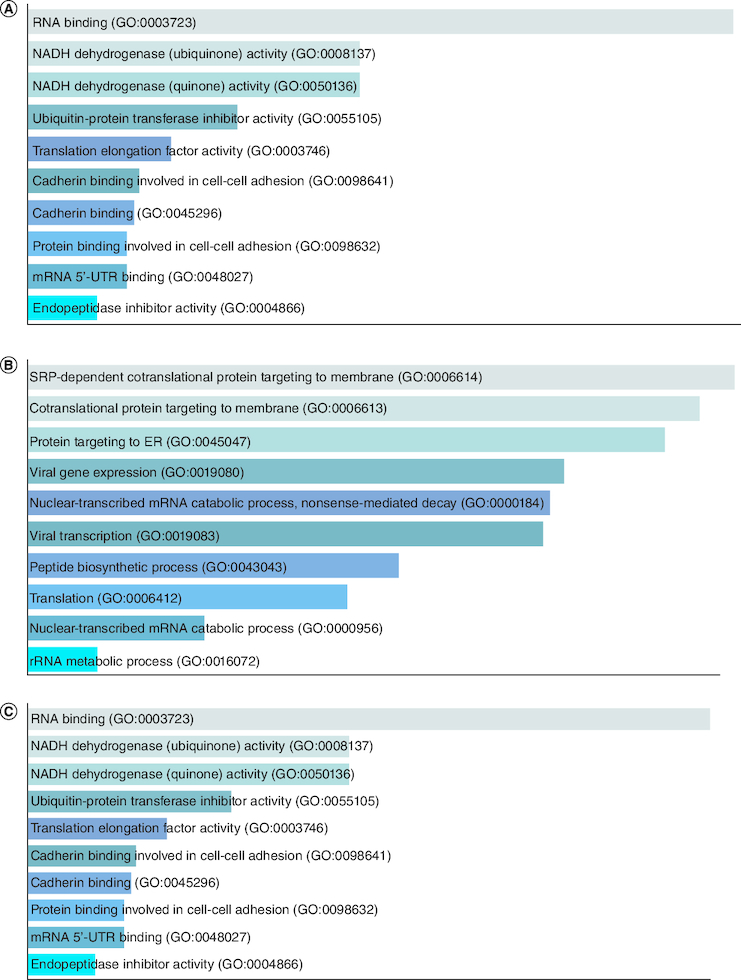
GO enrichment analysis of Group III under expressed genes in tumor data. **(A)** GO biological process, **(B)** GO molecular function and **(C)** GO cellular component.

Group III under expressed genes have 51 genes which have some members of keratin family and some are mitochondrial genes that are linked with electron transport chain there are also some ribosomal proteins. *RPS7* gene is a tumor suppressor protein, but its mechanism in cancer remains unclear. In the following study it has been shown that it inhibits colorectal cancer cell glycolysis, it has also been found that RPS7 gene regulates cell apoptosis and cell cycle. Mutations in *RPS7* gene have been shown to be associated with Diamond-Black fan Anemia (DBA) [45]. Overexpression of *CSTA* gene inhibits tumor cell growth, migration and invasion through regulating the MAPK and AKT pathways [46]. Figure 9A GO biological process, Figure 9B GO molecular function and Figure 9C GO cellular component.

### PPI network of genes

**Figure 10.**
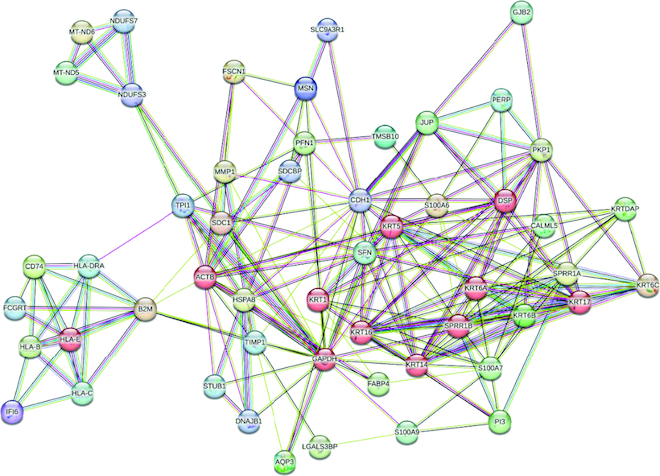
Protein protein interaction (PPi) of the over expressed groups are generated with the help of STRING online tool, the ones with red color indicated the hubs of the interaction between these groups.

Protein Protein Interaction (PPI) between the three over expressed genes. The results showed that *S100A9* gene of group I, is a calcium binding protein was the most overexpressed gene, *P13* and *MT-RNR2.* The ones with red color indicated the hubs of the interaction between these groups, which is also shown in Figure 10.

## Discussion

Through this finding, 51 up-regulated and 61 down-regulated genes out of 60483 transcripts in HNSCC data from TCGA data portal was found and performed a GO enrichment analysis of all the genes.

The group I over expressed genes have members of keratin family and S100A9 these genes are required in the maintenance of cellular damage and are highly produced when the cell is under stress conditions this can be the cause of over expression of these genes in tumor tissues [21]. Due to stress in a cell *KRT14* is produced and if cellular damage occurs because of the stress, *S100A9* is produced as immune response and for the maintenance and healing of wounds *KRT16 and KRT6A* is highly expressed [32].

The group II over expressed genes also had members of the keratin family, *P13, S100A7* and *MT-RNR2.* The cause of over expression of keratin family and *S100* family is that because they are involved in stress and maintenance of the cell. Whereas *P13* gene and its isoforms induce apoptosis but *MT-RNR2* suppresses apoptosis [37], which is the opposite of *P13* gene. This can be the reason that they are expressed similarly.

Group III has a large set of genes and plays significant roles in cells, noteworthy ones included ACTB which regulates transcription and repair DNA damage, SFN which is involved in epithelial cell growth [40], SLC2JA5 which exchanges cytoplasmic ADP to mitochondrial ATP, FABP4 which transports long-chain fatty acids, MT-ND5 which transfer electrons from NADH to the respiratory chain, HLA-C and HLA-B which are cell surface antigens, *HSPA8* which is expressed due to stress in the cell, and AQP3 which is a water channel. All these functions indicate that these genes are over expressed because they are primarily involved in glycolysis, transport, cell mobility and in tumor transformation, the following pathways may help cancer tissues grow and spread in the body. Protein protein interaction (PPi) is shown between the three over expressed in Figure 9.

In group I under expressed genes we have two genes *KRT4 and KRT13* these are found in differentiated layers of the mucosal and esophageal epithelia, these genes give instruction to produce keratin proteins that form the structural framework of epithelial cells but due to mutations in these genes they are under expressed [41].

Group II under expressed genes are involved in transformation of electrons from NADH to the respiratory chain and in apoptotic cell death. The reason they were under expressed in tumor tissue maybe because of mutation or drugs considered for treatment of cancer [44,47].

Group III also has a large set of genes, many genes from this group is involved in anti-inflammatory activities, microtubule stabilization and has antitumor activities, but might be because of mutations in these genes they were under expressed in tumor tissues due to which tumor was developed [45].

## Conclusion

In conclusion, the up-regulation of genes associated with stress and cellular maintenance in the current study suggests a direct link between the exposure to harmful environmental factors and subsequent cellular damage, potentially leading to oncogenesis. Smokeless tobacco products, poor oral hygiene, and chronic inflammation have been identified as potential risk factors that contribute to the accumulation of genetic mutations that may contribute to the development of cancer. The S100A9 gene of group I, is a calcium binding protein was the most overexpressed gene, P13 and MT-RNR2. It is crucial to raise awareness about the detrimental effects of these environmental factors and promote healthy lifestyle choices to reduce the incidence of cancer and other associated health risks. Further research is needed to develop targeted prevention and treatment strategies to combat the development and progression of cancer associated with environmental risk factors. Apply this methodology on all 33 cancer types available on TCGA. Identification of specific inhibitors, which can aid future drug discovery efforts for the cancer. Check the interactions of these genes with available drugs.
